# Characterization of HIV-1 envelopes in acutely and chronically infected injection drug users

**DOI:** 10.1186/s12977-014-0106-8

**Published:** 2014-11-28

**Authors:** Behzad Etemad, Oscar A Gonzalez, Laura White, Oliver Laeyendecker, Gregory D Kirk, Shruti Mehta, Manish Sagar

**Affiliations:** Boston University School of Medicine, Boston, MA USA; Boston University School of Public Health, Boston, MA USA; Johns Hopkins University School of Medicine, Baltimore, MD USA; Division of Intramural Research, National Institute of Allergy and Infectious Diseases, National Institutes of Health, Bethesda, MD USA; Johns Hopkins University, Bloomberg School of Public Health, Baltimore, MD USA

**Keywords:** HIV-1, Envelope, Transmission, Receptor, Replication, Injection drug use, Dendritic cells, Selection, Interferon

## Abstract

**Background:**

Mucosally acquired human immunodeficiency virus type 1 (HIV-1) infection results from a limited number of variants, and these infecting strains potentially have unique properties, such as increased susceptibility to entry blockers, relative interferon-alpha (IFN-α) resistance, and replication differences in some primary cells. There is no data about the phenotypic properties of HIV-1 envelope variants found early after acquisition among subjects infected through injection drug use (IDU). For the first time, we compared the characteristics of virus envelopes among injection drug users sampled prior to seroconversion (HIV RNA+/Ab-), within 1 year (early), and more than 2 years (chronic) after estimated acquisition.

**Results:**

Virus envelopes from 7 HIV RNA+/Ab- subjects possessed lower genetic diversity and divergence compared to 7 unrelated individuals sampled during the chronic phase of disease. Replication competent recombinant viruses incorporating the HIV RNA+/Ab- as compared to the chronic phase envelopes were significantly more sensitive to a CCR5 receptor inhibitor and IFN-α and showed a statistical trend toward greater sensitivity to a fusion blocker. The early as compared to chronic infection envelopes also demonstrated a statistical trend or significantly greater sensitivity to CCR5 and fusion inhibitor and IFN- α. The HIV RNA+/Ab- as compared to chronic envelope viruses replicated to a lower extent in mature monocyte derived dendritic cells – CD4+ T cell co-cultures, but there were no significant replication differences in other primary cells among the viruses with envelopes from the 3 different stages of infection.

**Conclusions:**

Similar to mucosal acquisition, HIV-1 envelope quasispecies present in injection drug users prior to seroconversion have unique phenotypic properties compared to those circulating during the chronic phase of disease.

**Electronic supplementary material:**

The online version of this article (doi:10.1186/s12977-014-0106-8) contains supplementary material, which is available to authorized users.

## Background

Although injection drug use (IDU) is a relatively common mode of HIV-1 acquisition, only a small number of studies have examined genotypic properties of the viruses found in newly infected subjects who presumably acquired the infection through IDU [1-4]. Furthermore, phenotypic characteristics of the viruses circulating in these newly infected individuals have not been examined in detail. Transmitted-founder (T/F) or viruses isolated prior to seroconversion have been most extensively studied in individuals who acquired their infection through sexual contact [5-14]. There are significant differences in acquiring HIV-1 sexually compared to from IDU. Sexual acquisition is relatively inefficient potentially because the virus must cross a mucosal barrier to infect early target cells at the site of invasion prior to establishing a systemic infection. On the other hand, the estimated frequency of transmission is much higher during IDU compared to the various modes of sexual contact, potentially because infectious virus often has direct access to bloodstream susceptible cells [15]. Sexual HIV-1 acquisition is also associated with a selective bottleneck during transmission [1,4,6-9,16-20]. Only a limited number of variants, sometimes only one, successfully establish an infection in a naïve host even though the transmitting partner harbors a diverse range of viruses. Interestingly, we and others have shown that HIV-1 infected injection drug users also often harbor a relatively limited number of viruses early in infection, although a larger number of variants often infect injection drug users compared to those who acquired the virus through sexual contact [1-3]. Because greater initial viral diversity is associated with faster disease progression [17], it is possible that characteristics of the infecting quasispecies are likely different among injection drug users compared to individuals who acquire the virus through sexual contact.

Diverse lines of evidence suggest that the observed bottleneck during sexual transmission occurs as a consequence of active selection rather than a stochastic process. Infecting viruses are often more closely related to HIV-1 variants found earlier during infection (termed ancestral strains) as compared to those circulating near the estimated time of transmission in the transmitting partner, which suggests the preferential selection of archived rather than contemporaneous strains during transmission [5,20-23]. In agreement with this genotypic observation, infecting viruses generally have smaller and less glycosylated envelopes compared to the dominant strains in the transmitting partner or variants isolated during chronic infection [20,24]. Because viruses expand their envelope length and increase the number of predicted glycosylation sites over the course of infection, this argues that viruses with genotypes closer to ancestral strains are favored for transmission [13,24-27]. The observation that newly infected subjects are predominantly infected with viruses that use the CCR5 receptor (termed R5) even though the transmitting partners often harbor both R5 and variants that can only use the CXCR4 receptor (termed X4) further suggests active selection during transmission [5,8,11,12,14,20,21,28,29]. In addition, viruses found during the chronic phase as compared to those circulating early after acquisition have decreased sensitivity to CCR5 inhibitors, suggesting they have an enhanced ability to use low levels of or structurally variant forms of the CCR5 receptor [30-34]. Together, this implies that chronic stage viruses that can only utilize the CXCR4 receptor or infect cells that have low levels or different conformations of the CCR5 receptor are not favored for transmission. Furthermore, recent studies demonstrate both that T/F as compared to chronic infection strains replicate to higher titers in the presence of IFN-α and viruses become more susceptible to IFN-α within 1 year after acquisition [11,35]. This implies that chronic stage variants with decreased replication in the presence of IFN-α have a disadvantage during transmission. In aggregate, these findings strongly suggest that the transmitted viruses with genotypic and phenotypic characteristics similar to ancestral strains have preferential advantage in establishing a systemic infection in a naïve host. It remains unclear if similar active selection occurs among viruses acquired through IDU as observed during sexual acquisition. In this study, we compared properties of the envelope quasispecies isolated from injection drug users sampled prior to HIV-1 seroconversion to those present over the first 2 to 3 years after acquisition.

## Results

### Subjects

We previously compared some characteristics among the virus envelope swarm present within 1 year (early) to those present around 2 – 3 years (chronic) after estimated seroconversion [34]. We wished to compare more envelope characteristics among these participants to unrelated subjects who also reported IDU and who were sampled prior to seroconversion (HIV RNA+/Ab-). None of the subjects had received any anti-retroviral treatment. Among the HIV RNA+/Ab- subjects, we chose to examine envelope quasispecies as opposed to the predicted T/F strains because injection drug users have been shown to acquire a greater number of variants [1-3]. As previously argued [36], we presumed that the combination of virus envelopes within a swarm would better recapitulate the envelope phenotype of the infecting virus population. Thus, we did not isolate presumed T/F strains from the HIV RNA+/Ab- subjects. In addition, we did not select specific envelopes from the early and chronic infection samples because this selection would bias the study. As a result, we used the same envelope isolation and virus construction methodology among the HIV RNA+/Ab- as used previously for the early and chronic infection group [34]. We successfully amplified full-length envelopes from 8 of 10 cohort subjects retrospectively confirmed as sampled prior to HIV-1 seroconversion. We could not amplify envelopes from the remaining samples with various different primer sets even though they contained relatively high plasma virus levels (25,873 and 98,533 copies/ml). Inability to amplify HIV-1 from some individuals even though they contain high virus levels is similar to previous studies from our group and others potentially suggesting sample properties prevents full-length envelope recovery [5,17,37]. Pooled amplification products from 4 independent PCRs were placed within a NL4-3 backbone, and virus stocks were generated using previous methods shown to reconstitute the viral quasispecies present in the original sample [30,38,39]. Indeed in our previous study, we showed that virus stocks contained similar level of genetic diversity and types of envelope variants as that present in the original sample [5]. Seven of the 8 envelope quasispecies incorporated within NL4-3 yielded infectious virus stocks. We compared the envelope characteristics from these 7 HIV RNA+/Ab- subjects to those from 7 longitudinally sampled individuals whose pool of amplified envelopes incorporated into NL4-3 also yielded infectious virus stocks (Table 1). All subjects, except A4 were hepatitis C virus (HCV) antibody positive prior to the time of estimated HIV-1 seroconversion. This is a strong marker for IDU. Indeed, 11 of the 14 subjects, including A4, reported injecting drugs in the 6 months prior to estimated HIV-1 seroconversion.

**Table 1:** **Subject characteristics**

**Subject**	**Estimated PI** **(months)** ^**1**^	**Plasma virus level**	**CCR5** ^**2**^	**CXCR4** ^**3**^	**Tropism** ^**4**^
**A31**	**Ab-/** **VL+**	**154,** **381**	**12.7**	**13.83**	**R5/X4**
**A33**	**Ab-/** **VL+**	**1,** **031,** **929**	**9.7**	**<0.03**	**R5**
**A38**	**Ab-/** **VL+**	**31,** **174**	**10.8**	**<0.03**	**R5**
**A40**	**Ab-/** **VL+**	**14,** **422**	**14.9**	**<0.03**	**R5**
**A41**	**Ab-/** **VL+**	**1,** **289**,**549**	**13.3**	**<0.03**	**R5**
**A42**	**Ab-/** **VL+**	**2,** **285,** **517**	**12.6**	**<0.03**	**R5**
**A43**	**Ab-/** **VL+**	**499,** **713**	**12.3**	**<0.03**	**R5**
**A2**	**3.0**	**85,** **439**	**11.5**	**<0.03**	**R5**
**A4**	**5.0**	**2,** **158,** **680**	**1.9**	**14.3**	**R5/X4**
**A5**	**4.1**	**279,** **745**	**8.7**	**<0.03**	**R5**
**A17**	**4.9**	**40,** **634**	**14.6**	**<0.03**	**R5**
**A18**	**3.2**	**188,** **258**	**12.1**	**<0.03**	**R5**
**A23**	**4.9**	**56,** **189**	**10.6**	**<0.03**	**R5**
**A27**	**4.1**	**152,** **472**	**10.4**	**<0.03**	**R5**
**A2**	**21.4**	**30,** **996**	**19.1**	**<0.03**	**R5**
**A4**	**24.5**	**79,** **094**	**0.3**	**12.2**	**R5/X4**
**A5**	**27.2**	**137,** **661**	**11.1**	**<0.03**	**R5**
**A17**	**20.9**	**20,** **995**	**13.0**	**<0.03**	**R5**
**A18**	**27**	**12,** **299**	**11.6**	**<0.03**	**R5**
**A23**	**27.7**	**79,** **490**	**12.1**	**<0.03**	**R5**
**A27**	**27.1**	**17,** **668**	**9.8**	**11.91**	**R5/X4**

^**1**^Interval in months from the estimated date of seroconversion to the day of sample collection. Ab-/VL+: Sampled prior to seroconversion.

^2^P24 (ug/ml) from U87/CD4/CCR5 cells at day 4 post-infection.

^3^P24 (ug/ml) from U87/CD4/CXCR4 cells at day 4 post-infection.

^4^Tropism as determined on U87/CD4+/CCR5 and U87/CD4+/CXCR4 cells.

As expected, the HIV RNA+/Ab- (median log_10_ plasma virus copies per ml 5.7, range 4.2 – 6.4) compared to the chronic (median log_10_ 4.5 copies/ml, range 4.1 – 5.1, p =0.05) samples had higher plasma virus levels. Plasma virus levels in the HIV-1 RNA+/Ab- as compared to the early (median log_10_ 5.2 copies/ml, range 4.6 – 6.3, p =0.6) samples were not statistically different. Longitudinal plasma virus levels significantly decreased from early in disease to the chronic phase of infection (median log_10_ plasma virus copies per ml difference 0.4, range −0.1 – 1.4, p =0.04). These plasma virus level differences followed the expected pattern of high virus replication immediately after acquisition and a decrease to a viral set point during the chronic phase of disease.

### Envelope sequences

**Figure 1:**
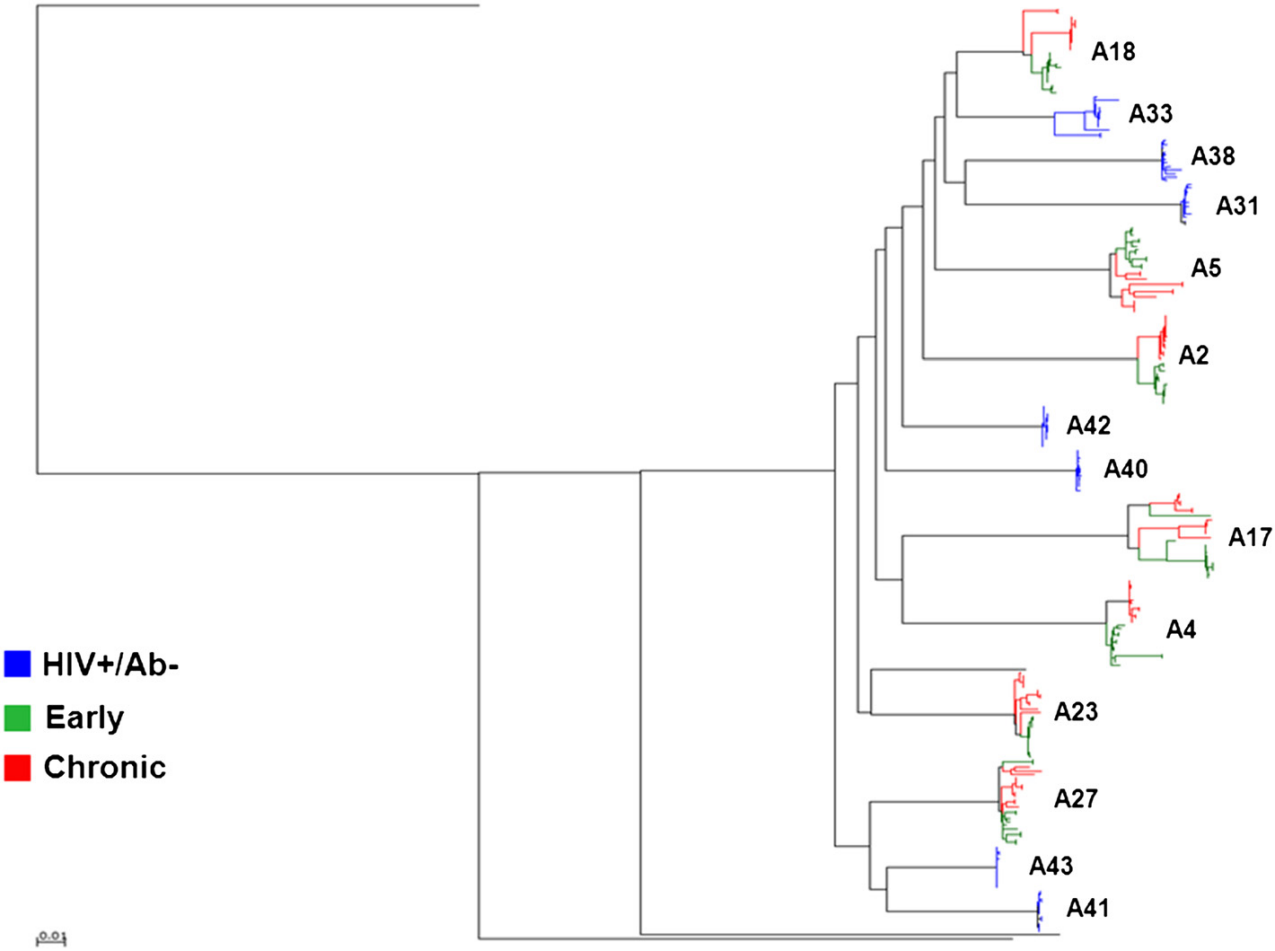
**The HIV RNA+/** **Ab-** **envelopes are not related to the virologically linked early and chronic infection variants.** Full-length HIV-1 envelope sequences were aligned with subtype reference sequences from the Los Alamos database using Clustal X. Maximum likelihood phylogenies were generated using Paup with parameters from FindModel best fit evolutionary model as described previously [21]. Subject IDs are noted with the different color nodes representing sequences from the 3 groups.

Unlike previous studies of individuals presumably infected through IDU, the primary goal of this study was not to examine genotypic features of the HIV-1 envelopes present at various times during infection [2,3]. Even though bulk PCR cloning as compared to single genome amplification (SGA) potentially introduces polymerase induced recombination artifacts, we examined 12 clonal sequences per sample because we compared overall virus population characteristics rather than individual genomes [6,40]. Phlyogenetic analysis confirmed that all subjects harbored subtype B HIV-1, and all early and chronic infection sequences were related ruling out contamination and HIV-1 superinfection (Figure 1). Envelopes from individuals sampled prior to seroconversion had lower genetic diversity (median 0.001, range 0.0004 – 0.01) compared to the chronic (median 0.007, range 0.001 – 0.02, p =0.05) group, but there were non-significant differences compared to envelopes from early (median 0.006, range 0.0008 – 0.008, p =0.2) infection. The HIV RNA+/Ab- envelopes (median 0.001, range 0.0005 – 0.01) were also less divergent than the chronic (median 0.007, range 0.001 – 0.02, p =0.05) and not statistically significantly different compared to the early (median 0.004, range 0.0007 – 0.02, p =0.2) infection genotypes. Similar to previous publications that examined HIV-1 subtype B envelope genes by the bulk PCR cloning methodology, the 3 groups did not have significant differences in envelope variable loop length or number of predicated aspargine linked glycosylation sites [24,27].

### Replication competent viruses

Bulk PCR envelope products were incorporated into a NL4-3 HIV-1 backbone to generate replication competent recombinant viruses using previously described methods [34]. Because we were interested in comparing the properties of the quasispecies at various times after infection, we examined envelope pools as opposed to individual SGA amplified envelopes. To confirm that potential polymerase induced recombination during bulk PCR did not generate novel phenotypes that did not exist in the original sample, we compared virus stocks containing a pool of envelopes generated using either SGA or bulk PCR. In 6 cases, the virus stocks with the bulk PCR and SGA envelope pools demonstrated equivalent infectivity and replication kinetics (Additional file 1: Figure S1). This suggests that the method for generating envelope pools did not have a significant influence on their *in*-*vitro* phenotypic properties.

### Receptor usage

**Figure 2:**
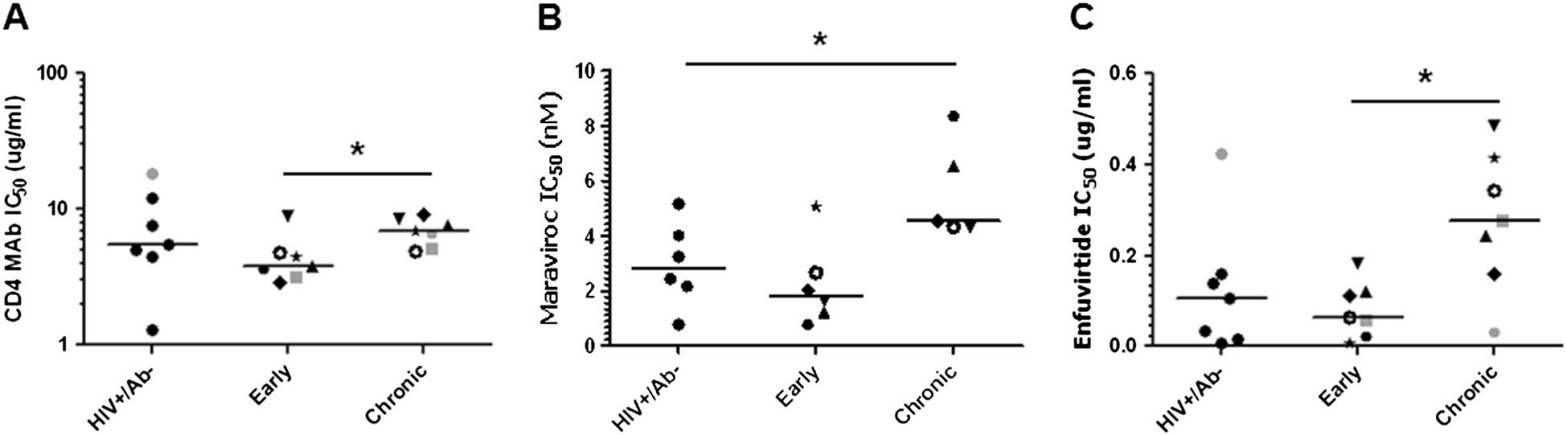
**The HIV RNA+/** **Ab-** **envelopes have greater sensitivity to CCR5 and fusion inhibitors.** Scatter plots show IC_50_s to CD4 antibody **(A)**, Maraviroc **(B)**, and Enfuvirtide **(C)** with a line denoting the median. Each individual symbol represents a subject’s mean from a minimum of 3 independent experiments. Circles denote the estimates for the HIV RNA+/Ab- subjects. The longitudinal sampling is denoted by a unique symbol (♦ A2, ■ A4, ○ A5, ★ A17, ▲ A18, ▼ A23, ●A27 ) for each subject. Symbols in gray denote virus stocks shown to be able to utilize the CXCR4 receptor. Significant differences (p ≤0.05) are indicated with a star in the graphs. Four envelope quasispecies with the documented ability to use the CXCR4 receptor were omitted from the examination of Maraviroc sensitivity.

*In*-*vitro*, the majority of HIV RNA+/Ab-, early and chronic infection envelopes used the CCR5 receptor and failed to employ the CXCR4 co-receptor (Table 1). We assessed sensitivity to receptor and fusion inhibitors as a way to examine if the envelopes from the various stages of infection had differences in their fusion capacity or ability to use low levels of CD4 and CCR5. We have previously shown that these inhibitor sensitivity assays clearly distinguish envelopes that require high versus low receptor levels for host cell entry [30]. The HIV RNA+/Ab- (median IC_50_ 5.6, range 1.3 – 18.1 ug/ml) as compared to the early (median IC_50_ 3.8, range 2.9 – 8.9 ug/ml, p =0.5) and chronic (median IC_50_ 7.0, range 4.9 – 9.2 ug/ml, p =0.6) envelopes showed no significant difference in their sensitivity to a monoclonal anti-CD4 antibody (Figure 2A). Paired analysis showed that the early as compared to the chronic infection envelopes were marginally more sensitive to the monoclonal anti-CD4 antibody (median IC_50_ difference 2.5, range −0.3 – 6.3 ug/ml, p =0.05).

CCR5 usage efficiency was assessed by estimating sensitivity to the CCR5 inhibitor, Maraviroc. Envelopes with documented ability to use the CXCR4 receptor were omitted from this analysis. At the highest Maraviroc concentration tested (50 nM), all tested envelopes showed greater than 95% inhibition suggesting there were no resistant envelopes as observed in previous studies [33,41]. The HIV RNA+/Ab- envelopes (median IC_50_ 3.2, range 0.8 – 6.3 nM) were more sensitive to Maraviroc compared to the chronic (median IC_50_ 4.5, range 4.3 – 8.4 nM, p =0.03) but not early (median IC_50_ 1.8, range 4.3 – 8.4 nM, p =0.3) variants (Figure 2B). Early envelope viruses also showed a trend toward increased Maraviroc sensitivity compared to the paired chronic infection variants (median difference 2.7, range 1.7 – 7.6 nM, p =0.06). This suggests that envelopes prior to seroconversion as compared to those present at the chronic phase require higher CCR5 receptor levels for cell entry.

Sensitivity to Enfuvirtide was used to assess fusion capacity. There was a trend that HIV RNA+/Ab- envelopes (median 0.1, range 0.01 – 0.4 ug/ml) were more sensitive compared to the chronic (median 0.3, range 0.03 – 0.5 ug/ml, p =0.07) but not the early variants (median 0.07, range 0.006 – 0.2 ug/ml, p =0.9) (Figure 2C). The early envelopes were significantly more sensitive to Enfuvirtide compared to the longitudinally isolated chronic variants (median difference 0.2, range 0.01 – 0.4 ug/ml, p =0.01). Similar to the Maraviroc findings, early phase infection envelopes have the greatest susceptibility, and pre-seroconversion as compared to the chronic phase envelopes have higher Enfuviritide sensitivity.

### Replication in primary cells

To assess replication, we monitored both p24 antigen and infectious virus concentration as assessed on TZM-bl cells in the culture supernatants post infection. The p24 antigen and infectious virus concentration curves over time displayed similar morphology among the diverse viruses. The area under the replication curve (AUC) from the p24 antigen measurements was highly correlated to the AUC from the infectious virus estimation (ρ =0.7, p =0.002, Spearman rank correlation) suggesting that either method could be used to follow virus production (Additional file 1: Figure S2).

**Figure 3:**
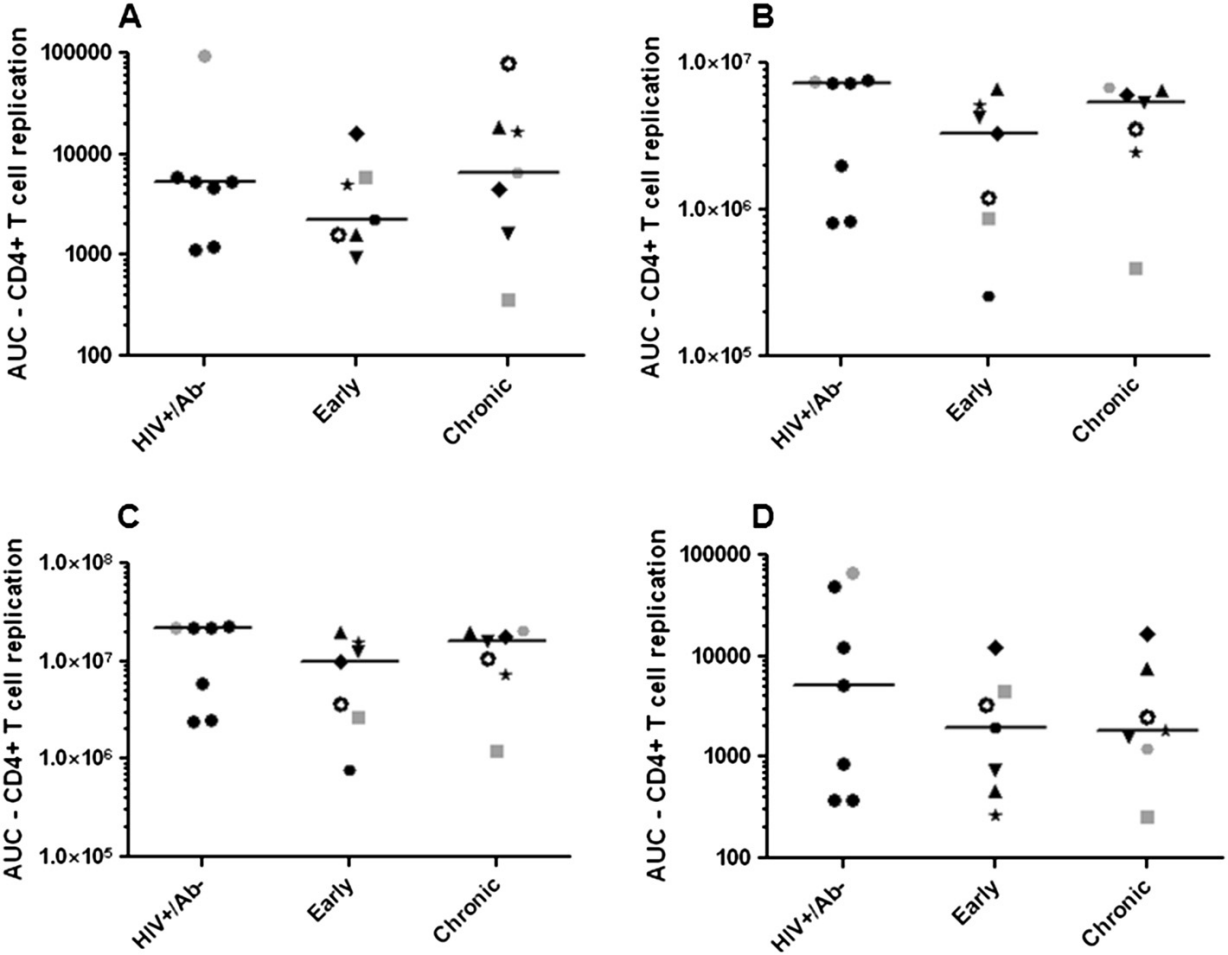
**Viruses with envelopes from different phases of infection demonstrate similar replication capacity in CD4+** **T cells.** Scatter plots show replication area under the curve (AUC) in activated CD4+ T cells from 4 different HIV-1 seronegative donors **(A-D)**. The AUC was calculated over 10 days post infection. Each individual symbol represents a subject’s AUC. Similar to Figure 2, in the early and chronic group unique symbols are used to show the longitudinal changes within an injection drug user. In each scatterplot, the lines denote the median. Symbols in gray denote virus stocks shown to be able to utilize the CXCR4 receptor.

Replication in primary cell cultures was used to examine if envelope quasispecies from different phases of infection confer varying replication capacity. Replication was examined in CD4+ T cells from 4 different individual donors. There was large replication variation between the different blood donor’s cells (Additional file 1: Figure S3). In addition, a virus stock that produced the highest area under the replication curve (AUC) in CD4+ T cells from 1 donor did not always yield the highest AUC in CD4+ T cells from other donors. Thus, different blood donation volunteers CD4+ T cells supported replication to varying levels in both individual virus stocks and among the entire group. As a result, we chose to analyze replication both individually in primary cells from 1 donor and in aggregate among primary cells from different donors. In cells from a majority of donors, the HIV RNA+/Ab- envelope viruses replicated to greater extent compared to the viruses with early and chronic infection envelopes, but these differences were not statistically significant in any of the donors individually (Figure 3). In aggregate, the HIV RNA+/Ab- envelopes also did not have statistically significant replication difference compared to the early (log estimated AUC difference 0.6, 95% confidence interval (CI) -0.1 – 1.3, p =0.07) or chronic (log AUC difference 0.1, 95% CI −0.7 – 0.8, p =0.7) group. The early infection envelope viruses did not have significantly different replication AUC compared to the chronic infection envelope variants in the CD4+ T cells, both in aggregate (log AUC difference 0.3, 95% CI −1.1 – 1.6, p =0.6) or when the cells from different donors were analyzed separately.

**Figure 4:**
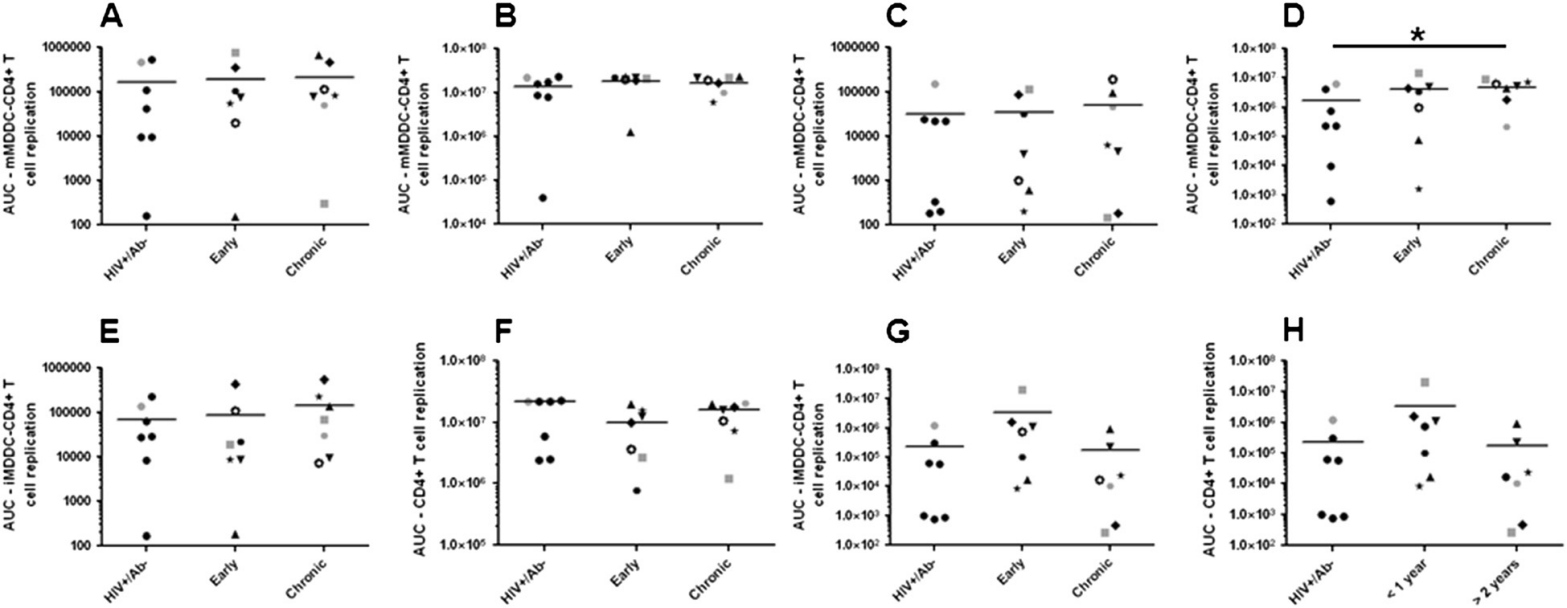
**Viruses with HIV RNA** **+/Ab-** **compared to chronic envelopes have lower replication in mature MDDC** **–** **CD4** **+** **T cells.** Similar to Figure 3, scatter plots show replication area under the curve in mature **(A – D)** and immature **(E – H)** MDDC – CD4+ T cell co-cultures from 4 different HIV-1 seronegative donors. Similar to Figure 2, in the early and chronic group unique symbols are used to show the longitudinal changes within an injection drug user. In each scatterplot, the lines denote the median. Significant differences (p ≤0.05) are indicated with a star in the graphs. Symbols in gray denote virus stocks shown to be able to utilize the CXCR4 receptor.

Dendritic cells (DCs) have been proposed to play an important role in HIV-1 transmission because they can efficiently capture viruses and transmit them to CD4+ T cells, which significantly enhances virus replication [42-44]. We examined if the envelope quasispecies from different phases of infection confer differences in ability to utilize monocyte derived DCs (MDDCs). Again, donor cell variability was also evident in MDDCs from different donors (Additional file 1: Figures S4 and S5). In 1 donor’s mature MDDCs – CD4+ T cell co-culture, the HIV RNA+/Ab- replicated less efficiently as compared to the chronic viruses (Figure 4). In aggregate, the HIV RNA+/Ab- envelope viruses had significantly lower replication AUC compared to the chronic (log AUC difference −1.3, 95% CI −2.6 – 0.002, p =0.03) but non-significant differences compared to the early (log AUC difference −0.7, 95% CI −2.1 – 0.6, p =0.3) infection envelopes in mature MDDC – CD4+ T cell co-cultures. There was, however, no statistical trend or significant difference among the early versus chronic envelopes (log AUC difference 1.5, 95% CI −1.3 – 4.2, p =0.3).

The HIV RNA+/Ab- envelopes also showed lower replication AUC compared to the early (log AUC difference −1.0, 95% CI −2.3 – 0.3, p =0.1) and chronic variants (log AUC difference −0.4, 95% CI −1.5 – 0.9, p =0.6) in immature MDDC – CD4+ T cell co-cultures, but the differences were not statistically significant. In aggregate, the early as compared to the chronic infection envelope viruses also did not have significant replication differences in immature MDDC – CD4+ T cell cocultures (log AUC difference −3.1, 95% CI −7.3 – 0.7, p =0.1).

### Gut homing receptor usage

**Figure 5:**
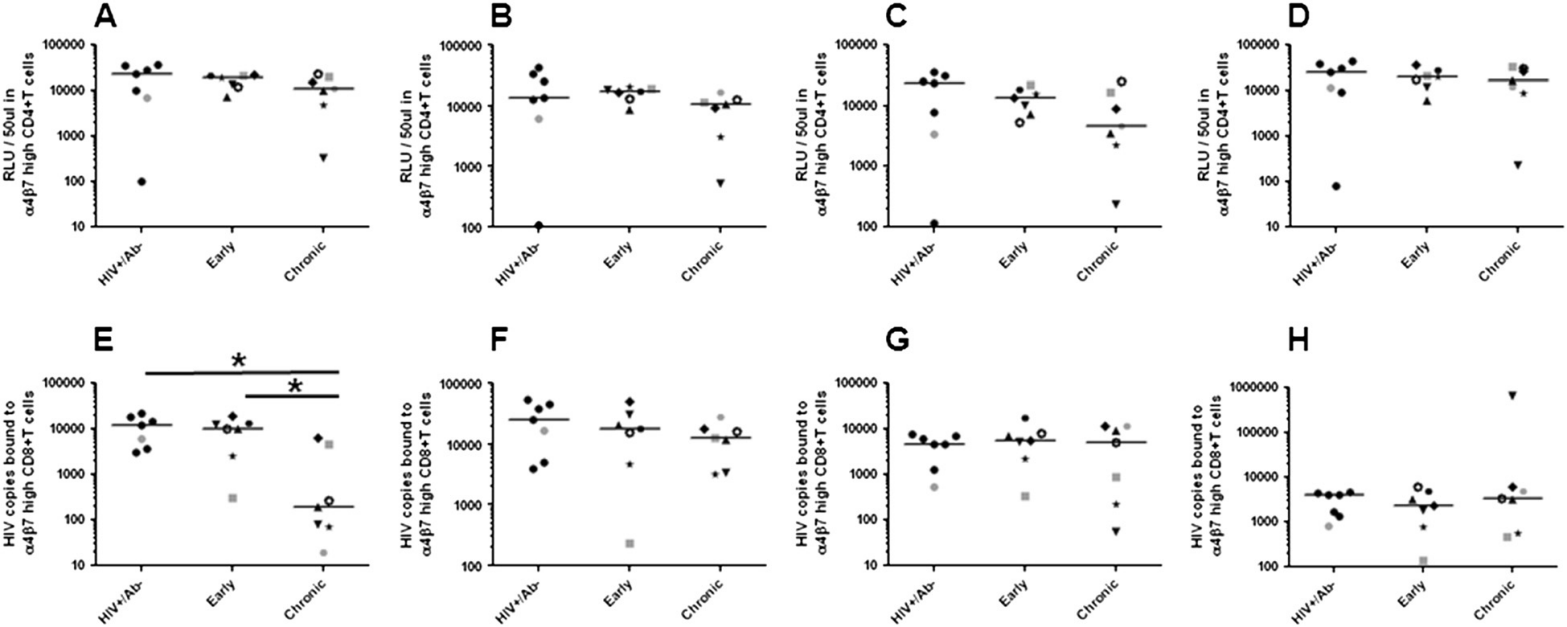
**Viruses with envelopes from different phases of infection demonstrate similar replication in α4β7 high CD4+** **T cells.** Scatter plots show relative light units in TZM-bl cells infected with 50 ul of virus supernatants recovered from virus exposed FACS confirmed α4β7 high CD4+ T cells **(A – D)**. Each symbol represents mean of 3 independent RLU measurements. Scatter plots show number of RNA copies recovered from virus exposed FACS confirmed α4β7 high CD8+ T cells from 4 different HIV-1 seronegative donors **(E - H)**. Each symbol represents mean of 2 independent RNA copy quantifications. Similar to Figure 2, in the early and chronic group unique symbols are used to show the longitudinal changes within an injection drug user. In each scatterplot, the lines denote the median. Significant differences (p ≤0.05) are indicated with a star in the graphs. Symbols in gray denote virus stocks shown to be able to utilize the CXCR4 receptor.

Early after HIV-1 acquisition, high level virus replication occurs in gut associated lymphoid tissue (GALT) [45]. The gut homing receptor, α4β7, potentially facilitates virus migration from the site of acquisition to the GALT [46-48]. In our previous studies, we showed binding and replication augmentation in cells with increased as compared to normal α4β7 expression among viruses with known α4β7 reactivity, such as HIV-1_SF162_ and HIV-1_Bal_ [49]. In addition, previous studies from our group and others have shown that α4β7 inhibitors often fail to prevent virus replication in and binding to cells expressing high levels of α4β7 [12,49]. Thus, we used similar methods to examine if envelope quasispecies from the 3 phases of infection had replication and binding differences in retinoic acid (RA) stimulated CD4+ and CD8+ T cells with flow cytometry confirmed high levels of α4β7 receptor respectively in the absence of any inhibitors. The HIV RNA+/Ab- envelopes showed no significant replication difference compared to the early (log RLU difference −0.5, 95% CI −1.3 – 0.2, p =0.1) or chronic (log RLU difference 0.3, 95% CI −0.6 – 1.2, p =0.5) envelopes in α4β7 high CD4+ T cells (Figure 5A – D). In addition, replication was not significantly different in the α4β7 high CD4+ T cells in any of the 4 donors when analyzed separately. Individually and in aggregate, the early as compared to the chronic infection envelope viruses also did not have significant replication differences in α4β7 high CD4+ T cells (log RLU difference 1.0, 95% CI −0.2 – 2.8, p =0.4).

The HIV RNA+/Ab- as compared to the chronic envelope viruses showed significantly greater binding to α4β7 high CD8+ T cells in 1 of the 4 donors (Figure 5E - H). In aggregate, the HIV RNA+/Ab- envelopes also showed significantly greater binding compared to the chronic (log RNA copy difference 1.0, 95% CI 0.02 – 1.9, p =0.03) but not the early (log RNA copy difference 0.3, 95% CI −0.3 – 1.0, p =0.3) envelopes. The early as compared to the chronic infection envelope viruses did not have significant binding differences to α4β7 high CD8+ T cells (log RNA copy difference 0.6, 95% CI −0.01 – 0.6, p =0.2), although significant difference was observed in 1 donor’s cells.

### Susceptibility to IFN-α

**Figure 6:**
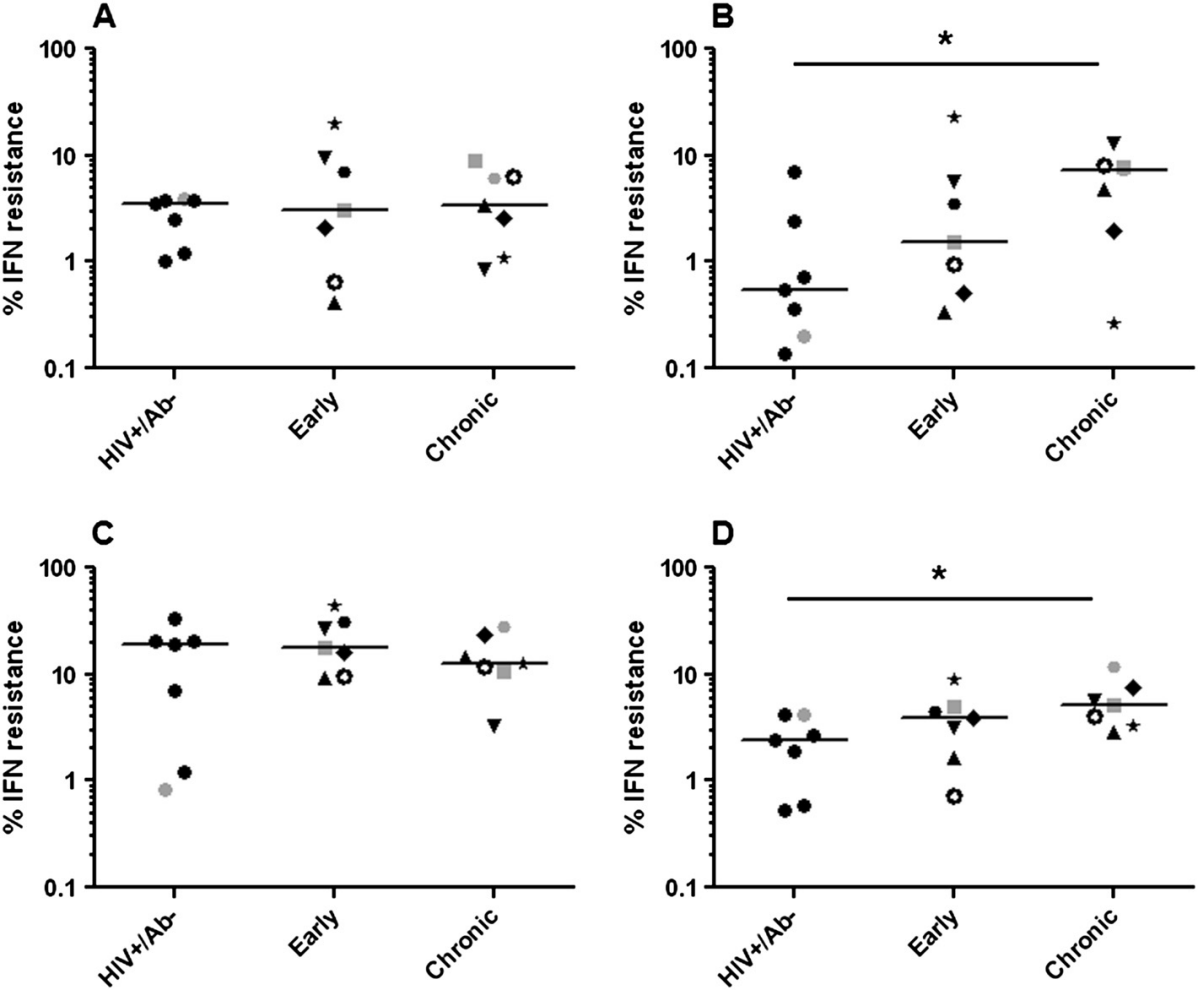
**The HIV RNA+/** **Ab-** **compared to the chronic envelopes have greater sensitivity to interferon-** **α.** Scatter plots show % interferon resistance (replication in the presence as compared to the absence of IFN- α) in CD4+ T cells from 4 different HIV-1 seronegative donors **(A – D)**. Similar to Figure 2, in the early and chronic group unique symbols are used to show the longitudinal changes within an injection drug user. In each scatterplot, the lines denote the median. Significant differences (p ≤0.05) are indicated with a star in the graphs. Symbols in gray denote virus stocks shown to be able to utilize the CXCR4 receptor.

In the SIV/macaque model, it has been shown that exposure to virus elicits a strong anti-viral response, such as elevated levels of IFN-α [50]. These anti-viral responses induce target cells, such as activated CD4+ T cells, to the site of invasion and also inhibit virus replication. Recent studies suggest that HIV-1 variants able to replicate in the presence of high interferon levels are potentially favored to establish an infection in a naïve host [11,35]. We examined if the virus stocks with HIV RNA+/Ab- envelope quasispecies demonstrated greater IFN-α resistance compared to the early and chronic infection envelope viruses (Additional file 1: Figure S6). Surprisingly, the HIV RNA+/Ab- envelope viruses demonstrated significantly lower resistance compared to the chronic group in CD4+ T cells from 2 of the 4 donors (Figure 6). In aggregate, the HIV RNA+/Ab- envelope viruses had a statistically significant 0.7 log lower %IFN-α resistance compared to the chronic group (95% CI −1.23 – -0.15, p =0.008). There was also a statistical trend that the HIV RNA+/Ab- envelopes were more IFN-α sensitive compared to the early group (log % IFN resistance difference −0.51, 95% CI −0.6 – 1.2, p =0.08). In aggregate, the early as compared to chronic infection envelope viruses showed a trend towards greater IFN-α sensitivity (log %IFN-α resistance difference −0.51, 95% CI −1.14 – 0.12, p =0.1). This suggests that in injection drug users IFN-α resistance increases from the pre-seroconversion to the early and eventually to the chronic phase of infection.

## Discussion

Even though IDU is a relatively common route for HIV-1 acquisition, there has been limited understanding about the pathogenesis associated with this mode of transmission. In contrast to mucosal acquisition, viruses acquired through IDU do not encounter an epithelial barrier in accessing potential target cells, suggesting that the characteristics of the acquired variants may be different among mucosal versus non-mucosal acquisition. Indeed, previous studies have suggested that injection drug users are often infected with more variants compared to individuals that acquire the virus through heterosexual or homosexual contact [1-3]. In this study, we primarily examined the phenotypic characteristics of the virus envelope quasispecies isolated prior to seroconversion, within an estimated 1 year after acquisition, and during the chronic phase of disease among individuals that presumably acquired HIV-1 through IDU. We found that the envelope quasispecies in HIV RNA+/Ab- compared to the chronically infected subjects were significantly more sensitive to a CCR5 inhibitor and IFN-α, and had a trend towards increased susceptibility to Enfuvirtide. Interestingly, the early as compared to the chronic infection envelope quasispecies displayed either a statistical trend or significantly greater sensitivity to Maraviroc, Enfuvirtide, and IFN-α. This suggests that injection drug users prior to seroconversion possess unique HIV-1 envelope quasispecies that require high CCR5 receptor levels for host cell entry, have lower fusion capacity, and confer enhanced sensitivity to IFN-α. Over the first 2 to 3 years of infection, virus envelope quasispecies evolve the ability to use lower CCR5 levels, have enhanced fusion capacity, and confer decreased IFN-α sensitivity. In aggregate, our findings suggest that viruses with unique envelope characteristics preferentially establish a systemic infection among injection drug users.

Previous studies from our group and others show that the genetic bottleneck during IDU transmission is not as restrictive because the likelihood of acquiring more than a single virus appears higher compared to other routes of HIV-1 acquisition, such as sexual contact [1-3]. Even though the genetic bottleneck is not as restrictive compared to acquisition across a mucosal barrier, the number of infecting variants is still less than what would be typically present in an individual sampled during the early and chronic phases of disease [51]. Smaller number of variants than expected may be circulating in newly infected injection drug users because the virus could be acquired from another individual in the acute phase of infection with relatively limited viral genetic diversity. On the other hand, contaminated needles may only harbor a small number of infectious virions. Because it is logistically difficult to sample the transmission source, it remains unclear if the relatively limited number of variants observed in injection drug users early after acquisition is due to active selection or limited source diversity.

We and others have previously shown that mucosally acquired viruses sampled early after acquisition, including T/F viruses, have decreased ability to use low levels of or different conformation of the CCR5 receptor compared to unrelated chronic controls [30,31,33]. In this study, we also observed that swarm present prior to seroconversion showed greater sensitivity to Maraviroc compared to the swarm sampled during the chronic phase of disease. Because CCR5 sensitivity is directly correlated to fusion inhibitor susceptibility [52], we also found that the HIV RNA+/Ab- envelope variants showed a trend toward greater sensitivity to Enfuvirtide compared to the chronic strains [30,52,53]. In aggregate, pre-seroconversion variants present in injection drug users and mucosally acquired strains require high levels of CCR5 in an invariant structure to enter host cells. This potentially suggests that similar biological mechanisms favor the ability of relatively CCR5 receptor and fusion inhibitor sensitive variants in establishing a disseminated infection regardless of whether the virus is acquired through IDU or across a mucosal barrier.

Interestingly, we found that envelopes isolated prior to seroconversion conferred greater sensitivity to IFN-α compared to the chronic stage envelopes. Two previous investigations have shown that full-length T/F and acute stage viruses are relatively interferon resistant [11,35]. This has been used to argue that viruses able to replicate in the presence of high interferon levels at the site of invasion preferentially establish a systemic infection leading to the observed genetic bottleneck during transmission. Interferon resistance may not lead to a genetic restriction in injection drug users because IDU is potentially associated with persistent inflammation [54,55]. Thus, when a naïve individual acquires HIV-1 from a chronically infected injection drug user interferon susceptibility will not present a barrier for most variants in establishing a new systemic infection because viruses circulating in the transmitter may already be relatively interferon resistant. Indeed, a previous study failed to show interferon susceptibility difference between sexually acquired subtype C T/F versus chronic phase variants presumably because the chronically infected transmitters have persistent inflammation [11]. In addition, while the observed differences were statistically significant, it remains unclear if they are clinically meaningful because, in aggregate, the chronic infection viruses were around 2 fold more resistant to interferon compared to the HIV RNA+/Ab- envelope variants. On the other hand, we may have failed to replicate the previous findings because our studies only examined envelope differences, and non-envelope portions of the virus genome may be the genetic determinant for interferon resistance [56].

We also found that the HIV RNA+/Ab- as compared to the chronic envelope viruses replicated less efficiently in mature MDDC – CD4+ T cells. This corroborates other published findings showing that chronic infection envelope quasispecies compared to unrelated sexually acquired T/F envelopes demonstrated higher replication in MDDC – CD4+ T cell co-cultures [39]. Furthermore, in our previous study, we found that viruses with envelopes from chronically infected individuals replicated to greater extent in mature MDDC – CD4+ T cells compared to the variants with envelopes from the newly infected heterosexual partner [5]. It should be noted that one report suggests that full-length T/F as compared to chronic infection controls have greater replication in MDDC – CD4+ T cell co-cultures although significant differences were only observed in subtype B and not subtype C HIV-1, and the results were based on infections in primary cells from only two different donors [11]. In summary, these results suggest that if dendritic cells play a potential role during initial HIV-1 acquisition then the envelope glycoprotein does not solely dictate which variants successfully establish a new infection.

Our findings failed to reveal replication differences in any of the other primary cells among viruses sampled during the various phases of disease. Even though, HIV RNA+/Ab- as compared to the chronic envelopes demonstrated significantly greater binding to α4β7 high CD8+ T cells, importantly, there was no significant replication difference in the α4β7 high CD4+ T cells. Replication as opposed to binding is likely the more important phenotype that determines a virus’ fitness for establishing a new infection in a naïve individual. Our previous study of recipient transmitter pairs showed that envelope variants circulating in the chronically infected sexual partner compared to those present in the newly infected subject had higher replication in CD4+ T cells with high or normal levels of α4β7 levels and with and without MDDCs [5]. In contrast, however, another study showed full length sexually acquired T/F strains had greater MDDC binding and trans infection compared to unrelated chronic infection controls [11]. There are likely a number of reasons we may have failed to observe replication differences in the other primary cells among the 3 groups in our study. Even though we detected some significant phenotypic differences, it is likely that we were statistically underpowered to detect small variations because we only examined 7 subjects in each group. Previous studies using relatively similar number of samples per group (n =6 to 11) showed significant differences in sensitivity to receptor inhibitors and replication kinetics among sexually acquired envelope variants compared to the unrelated chronic infection strains or variants circulating in the transmitting partner [5,14,39,57]. Because selection bottleneck is less restrictive during IDU compared to mucosal acquisition, it can be hypothesized that potential differences among the variants at various times post acquisition are likely smaller. Thus, larger sample sizes would be needed to document a significant difference. Although we obtained our samples from one of the largest cohorts of injection drug users [58], there are still only a limited number of samples from individuals sampled prior to HIV-1 seroconversion. We did not seek samples from other cohort of injection drug users because of subject heterogeneity and viral subtype differences [59,60]. In context to the statistical analyses, it should be noted that we did not adjust for multiple comparisons. Clearly conservative adjustments for multiple comparisons would have rendered all findings statistically insignificant given the small sample sizes. Regardless, the observed significant differences are unlikely due random chance alone because of the similarity in the observed unique phenotypic characteristics among the earliest variants sampled from both sexually and IDU acquired viruses.

Another reason we may have failed to observe replication differences among the groups in our study is because we compared the envelope variants isolated prior to seroconversion to viruses sampled later in infection from unrelated subjects [5]. The limited variants that establish a systemic infection in a naïve host may harbor a phenotype that favors its acquisition compared to the swarm present in the transmission source. This selection property, however, may not necessarily be significantly different among the acquired variants and unrelated chronic infection controls. It should be noted, however, that we examined envelope properties in the context of an infectious clone, which is different from most studies that use 293T derived single cycle virus pseudotypes [12,14,28,57]. Besides the fact that single cycle viruses cannot be used to examine replication in primary cells, 293T as compared to peripheral blood mononuclear cell (PBMC) derived viruses often have different envelope density and glycans, which can influence receptor binding, neutralization and other properties [47,61,62]. Thus, peripheral blood mononuclear cell (PBMC) generated viruses have more physiologically relevant phenotypes compared to the 293T transfection derived virions. Although, it cannot be unequivocally stated that PBMC passage does not affect the virus phenotype, changes are generally less likely to occur in the short term cultures (maximum 7 days) used in our study. In addition, because all virus stocks were generated in a similar manner, changes induced by the short term PBMC passage should have affected the viruses from the 3 different phases of infection in an equivalent manner.

There are a number of limitations with our study. First, similar to all investigations of injection drug users, one can never be certain that the subjects acquired their infection through IDU. Even though individuals may report active IDU and have strong markers for this activity, such as co-infection with HCV, there is no way to definitively exclude the possibility that they acquired their virus through sexual contact. Second, we only examined properties of the envelope glycoprotein quasispecies. It is quite possible that other viral genomic regions, such as gag, influence replication especially in MDDC – CD4+ T cell co-cultures [63,64]. Third, we did not isolate the T/F envelopes from the HIV RNA+/Ab- subjects. Although HIV-1 diversifies relatively quickly, there were likely minimal changes in the quasispecies isolated prior to seroconversion as opposed to the predicted T/F strains. In aggregate, from our studies, we can conclude that IDU acquired pre-seroconversion as compared to chronic phase infection virus envelope quasispecies when inserted into an isogenic backbone require higher CCR5 receptor levels, have lower fusion capacity, replicate less efficiently in MDDC-CD4+ T cell co-cultures, and confer enhanced IFN-α sensitivity.

## Conclusion

To our knowledge, this is the first study that has extensively examined the envelope phenotypic properties of HIV-1 variants found among individuals who presumably acquired their infection through IDU. A strength of this study is that we sampled subjects prior to seroconversion, and thus we were able to characterize the earliest virus swarm after acquisition, albeit not the predicted T/F variants. While previous genotypic studies suggest that limited number of variants, although likely greater than the number acquired across mucosal surfaces, establish a systemic infection in injection drug users, our phenotypic data suggest that these earliest envelopes have unique envelope properties compared to chronic infection variants. Phenotypic similarities among mucosally and IDU acquired viruses, such as a requirement for high CCR5 receptor levels, suggest that the different modes of acquisition share similar biological mechanisms that dictate the types of variants that establish a systemic infection in a naïve individual.

## Methods

### Subjects

All subjects examined were from the AIDS Linked to the IntraVenous Experience (ALIVE) cohort, which follows HIV-1 uninfected and HIV-1 infected injection drug users in Baltimore, Maryland through semiannual visits [58]. Estimated acquisition interval was based on serological testing of longitudinal samples. Newly seropositive subjects’ previously seronegative sample was tested for HIV-1 RNA with a pooled viral load assay as previously described to identify the HIV-1+/Ab- individuals [65,66]. The seroconversion date was estimated as the midpoint between the last HIV-1 seronegative visit and the day the first HIV-1 seropositive sample was obtained. All subjects sampled within a year (early) and around two to three after seroconversion (chronic) have been described previously [34]. The study was approved by human subjects review boards at Johns Hopkins University, Bloomberg School of Public Health, and Brigham and Women’s Hospital; all participants provided informed written consent.

### Envelope amplification and analysis

For all subjects, HIV-1 RNA was isolated from around 100 ul of the serum samples, and RT-PCR was used to amplify a library of full-length envelope genes using previously described primers and amplification conditions [6]. For each subject, a minimum of 4 independent PCRs were pooled to generate a library of envelopes from each serum sample. Pooled envelope amplifications were inserted into linearized pCMV-NL4-3-PBS → LTRΔGp160 plasmid using yeast gap-repair homologous recombination as previously described [34,38]. Clone pools were transfected into HEK293T cells and culture supernatants were passaged on PBMC for a maximum of 7 days. In contrast to our previous study [34], all virus stocks were generated from a combination of supernatants obtained from 3 independent cloning attempts from 3 different PBMC cultures. The number of infectious particles (IP) was estimated on TZM-bl cells as previously described [25,67].

Twelve individual full-length envelopes were isolated and sequenced from each subject’s clones. All unique sequences reported in this publication have been submitted to Genbank (accession numbers KP171242 - KP171495). Average of pairwise distances was used to estimate genetic diversity. Divergence was estimated as the average distance from the subject’s sequences to the estimated ancestor as described previously [21]. Amino acid lengths and number of predicted N-linked glycosylation sites (PNGS) of different envelope segments were analyzed as previously described [21].

### Inhibitor sensitivity

TZM-bl, U87/CD4/CXCR4 and U87/CD4/CCR5 cells, Enfuvirtide, Maraviroc, and CD4 B4 monoclonal antibody were obtained through Research and Reference Reagent Program, Division of AIDS, NIAID, NIH [68-70]. Infection of TZM-bl cells in the absence and presence of two-fold serial dilution of the inhibitor was used to estimate the 50% inhibitory concentration (IC_50_) as previously described [30]. All reported IC_50_s are mean estimates from a minimum of 3 independent assays. Coreceptor usage was determined by monitoring p24 production in U87/CD4/CXCR4 and U87/CD4/CCR5 cells infected with 500 IP of each virus supernatant.

### Primary cells and infections

PBMCs were isolated from HIV-1 negative blood donation volunteer’s buffy coats using Ficoll Hypaque density centrifugation. Monocytes were isolated from PBMCs using the percoll gradient method [71]. Primary human immature DCs were derived from monocytes, as described previously [72]. Briefly, monocytes were cultured in RPMI/10% FBS containing recombinant human GM-CSF (0.5 μg/ml; Leukine, Berlex) and recombinant human IL-4, 100 U/ml (Peprotech) for 6 days. Mature DCs were obtained by culturing immature DCs at day six of culture for two additional days in the presence of 100 ng/ml of ultra-pure *E. coli* LPS (Sigma). Primary human CD4+ and CD8+ T cells were positively isolated from monocyte depleted PBMCs using antibody conjugated magnetic beads (Miltenyi Biotech) according to manufacturer’s instructions. CD4+ T cells were activated with 2% phytohaemagglutinin (PHA) and 20 ug/ml recombinant human IL-2 (r-IL-2) for 2 days.

Around 2 × 10^6^ CD4+ T cells were exposed to 1,000 infectious particles in the presence of 20 U/ml diethylaminoethyl (DEAE) Dextran. After two hours, cultures were washed a minimum of three times to remove unbound virus. Around 0.5 × 10^6^ immature or mature DCs were independently exposed to 1,000 infectious particles. After three hours, DC cultures were washed a minimum of three times to remove unbound virus. Virus exposed DC infections were cultured either with or without autologous activated CD4+ T cells. Infectious virus concentration was estimated by infecting 1 × 10^4^ TZM-bl cells with 4 to 8 serial two-fold dilutions of supernatant culture starting at 50 ul (Additional file 1: Figures S6 and S7 for representative examples). All infections were done in triplicate in a 96 well format. Two days post-infection, TZM-bls were examined for beta-galactosidase production using Galacto-Light Plus System (Applied Biosystems). Virus stock dilutions in the non-linear range of the TZM-bl assay were discarded. A linear interpolated curve of the relative light units (RLUs) versus supernatant dilution was used to estimate RLU/ul. The AUC was generated from the plot of RLU/ul versus days post infection. Primary cell infections were repeated a minimum of 4 times with cells from 4 different buffy coats. Culture supernatants were also assessed for p24 antigen content using an in house assay as previously described [73].

### Replication in CD4+ and binding to CD8+ T cells expressing high α4β7 integrin levels

Gut homing receptor, α4β7, usage was examined as previously described [49]. Briefly, both CD8+ and CD4+ T cells were activated with PHA, r-IL-2, and retinoic acid (RA) for 6 days. Only cells confirmed to have enhanced α4β7 expression as determined by binding of phycoerythrin (PE) conjugated anti-mouse integrin β7 antibody (clone FIB27) (BioLegend) were used in the subsequent assays. Around 1 × 10^6^ CD8+ and CD4+ T cells were exposed to 1 × 10^5^ infectious virus for 1 hour at 4°C in HEPES-buffered saline with 100 μM CaCl_2_ and 1 mM MnCl_2_. Cells were washed a minimum of 3 times to remove unbound virus. RNA was isolated from the CD8+ T cells using the QIAAMP Viral RNA kit (QIAGEN). HIV-1 copies were quantified using quantitative RT-PCR using previously described methods [74,75]. The CD4+ T cells were incubated at 37°C 5% CO_2_, and the infectious virus concentration in the culture supernatants was measured after 3 days as detailed above.

### Replication in the presence of IFN-α

CD4+ T cells were pre-treated with 1000 U/ml IFN-2α (PBL Assay Science) for 4 hours. Around 2 × 10^5^ pre-treated and untreated CD4+ T cells were exposed to 2 × 10^3^ infectious virus for 2 – 3 hours. Following exposure, cells were washed and re-plated in the presence or absence of IFN-α along with 20 U/ml r-IL-2. After 7 days, supernatants were removed, and TZM-bl cells were exposed to 4 different supernatant dilutions. Importantly, we confirmed that TZM-bl infections were not affected by the presence of IFN-α (Additional file 1: Figure S7). The RLUs generated from the TZM-bl infections were measured 2 days after exposure, and the RLU versus supernatant dilution plot was used to estimate an AUC for each virus in the presence and absence of IFN-α. The % IFN-α resistance was estimated from the ratio of AUC in the presence compared to the absence of IFN-α.

### Statistical analysis

Summary characteristics were compared among the HIV RNA+/Ab- envelopes to the other group of viruses using the Wilcoxon rank-sum test. Longitudinal comparisons among the early and chronic samples were done using the matched pair Wilcoxon rank-sum test. All p-values were based on a two-sided test. Initially, comparisons were done independently for primary cells obtained from different donors. Linear regression models were used for the aggregate comparisons among the three groups in the primary cells from four different donors. The differences between chronic and early values were the outcome for the longitudinal analysis. Linear regressions models of the log transformed values were adjusted for the four different donors and considered an interaction between the three groups and the origin of the primary cells. The correlation between observations from the same donor was negligible, and adjusting for this via generalized estimating equations (GEE) had no impact on the results. Nonparametric bootstrapping with 1999 bootstrapped samples was used to obtain 95% confidence intervals. All statistical analyses were done with either Intercooled Stata version 8.0 (Stata Corporation, College Station, TX) or R 2.15 (r-project.org).

## Additional file

Additional file 1: Figure S1. Infectivity (I) and replication kinetics are not significantly different among recombinant viruses with envelopes amplified using either single genome amplification (SGA) or multiple bulk PCR. **Figure S2.** Virus replication kinetics are similar as measured by the detection of p24 and infectious virus on TZM-bl cells. **Figure S3.** Virus replication varies in CD4+ T cells from different donor. **Figure S4.** Virus replication varies in mature MDDC - CD4+ T co-cultures with cells from different donors. **Figure S5.** Virus replication varies in immature MDDC - CD4+ T co-cultures with cells from different donors. **Figure S6.** Virus replication in the presence or absence of IFN-α in CD4+ T cells from different donors.
