# Supplementary material for: Characterization of HIV-1 envelopes in acutely and chronically infected injection drug users
Source: Retrovirology. 2014 Nov 28;11:106. doi: 10.1186/s12977-014-0106-8 (PMC4253609; doi:10.1186/s12977-014-0106-8)
Supplement: Additional file 1: Figure S1. — Infectivity (I) and replication kinetics are not significantly different among recombinant viruses with envelopes amplified using either single genome amplification (SGA) or multiple bulk PCR. Figure S2. Virus replication kinetics are similar as measured by the detection of p24 and infectious virus on TZM-bl cells. Figure S3. Virus replication varies in CD4+ T cells from different donor. Figure S4. Virus replication varies in mature MDDC - CD4+ T co-cultures with cells from different donors. Figure S5. Virus replication varies in immature MDDC - CD4+ T co-cultures with cells from different donors. Figure S6. Virus replication in the presence or absence of IFN-α in CD4+ T cells from different donors. [file 12977_2014_106_MOESM1_ESM.pptx]

## Slide 1
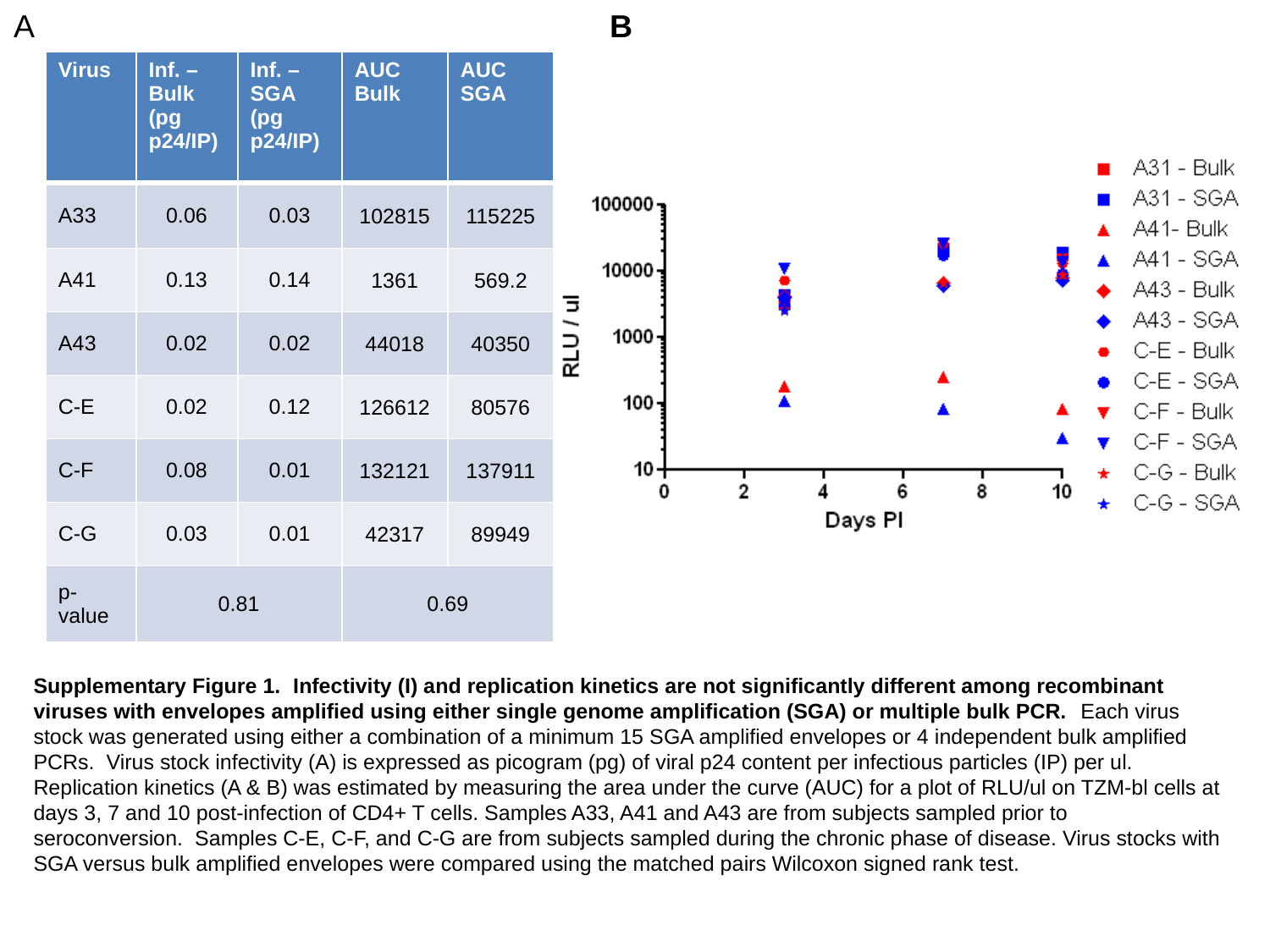

A
B
| Virus | Inf. –Bulk (pg p24/IP) | Inf. –SGA (pg p24/IP) | AUC Bulk | AUC SGA |
| --- | --- | --- | --- | --- |
| A33 | 0.06 | 0.03 | 102815 | 115225 |
| A41 | 0.13 | 0.14 | 1361 | 569.2 |
| A43 | 0.02 | 0.02 | 44018 | 40350 |
| C-E | 0.02 | 0.12 | 126612 | 80576 |
| C-F | 0.08 | 0.01 | 132121 | 137911 |
| C-G | 0.03 | 0.01 | 42317 | 89949 |
| p-value | 0.81 | | 0.69 | |
Supplementary Figure 1. Infectivity (I) and replication kinetics are not significantly different among recombinant viruses with envelopes amplified using either single genome amplification (SGA) or multiple bulk PCR. Each virus stock was generated using either a combination of a minimum 15 SGA amplified envelopes or 4 independent bulk amplified PCRs. Virus stock infectivity (A) is expressed as picogram (pg) of viral p24 content per infectious particles (IP) per ul. Replication kinetics (A & B) was estimated by measuring the area under the curve (AUC) for a plot of RLU/ul on TZM-bl cells at days 3, 7 and 10 post-infection of CD4+ T cells. Samples A33, A41 and A43 are from subjects sampled prior to seroconversion. Samples C-E, C-F, and C-G are from subjects sampled during the chronic phase of disease. Virus stocks with SGA versus bulk amplified envelopes were compared using the matched pairs Wilcoxon signed rank test.

## Slide 2
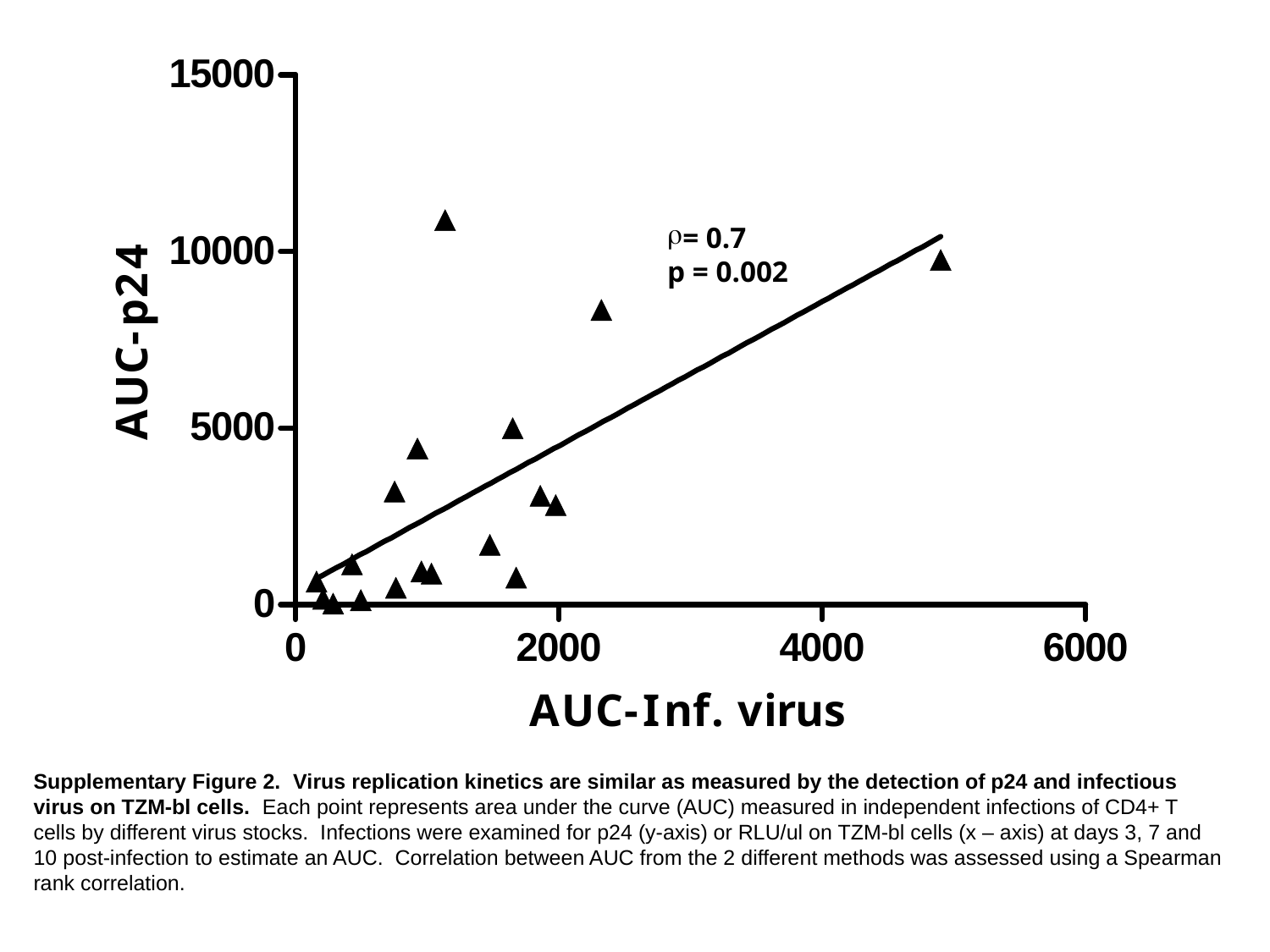

= 0.7
p = 0.002
Supplementary Figure 2. Virus replication kinetics are similar as measured by the detection of p24 and infectious virus on TZM-bl cells. Each point represents area under the curve (AUC) measured in independent infections of CD4+ T cells by different virus stocks. Infections were examined for p24 (y-axis) or RLU/ul on TZM-bl cells (x – axis) at days 3, 7 and 10 post-infection to estimate an AUC. Correlation between AUC from the 2 different methods was assessed using a Spearman rank correlation.

## Slide 3
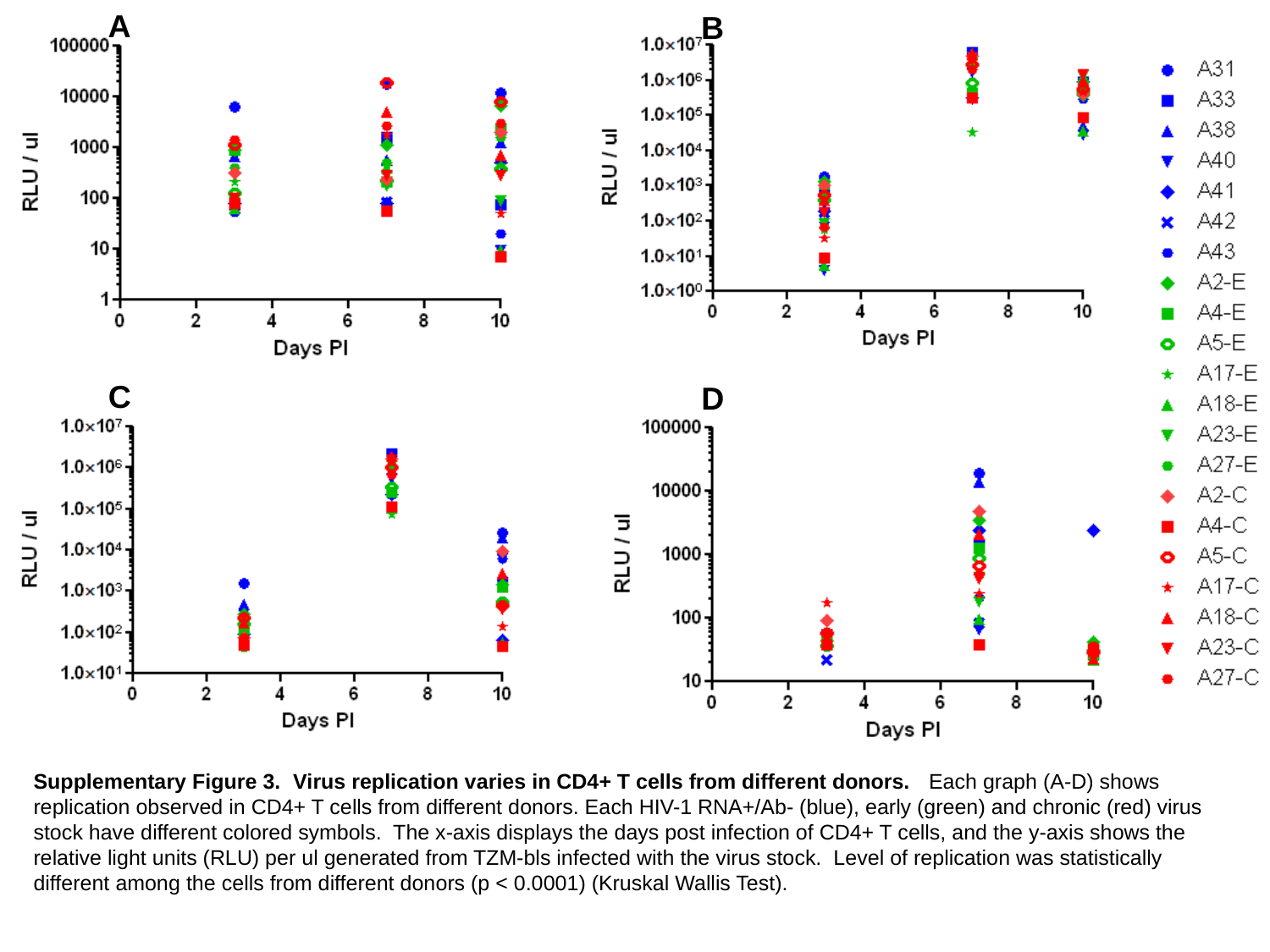

A
B
C
D
Supplementary Figure 3. Virus replication varies in CD4+ T cells from different donors. Each graph (A-D) shows replication observed in CD4+ T cells from different donors. Each HIV-1 RNA+/Ab- (blue), early (green) and chronic (red) virus stock have different colored symbols. The x-axis displays the days post infection of CD4+ T cells, and the y-axis shows the relative light units (RLU) per ul generated from TZM-bls infected with the virus stock. Level of replication was statistically different among the cells from different donors (p < 0.0001) (Kruskal Wallis Test).

## Slide 4
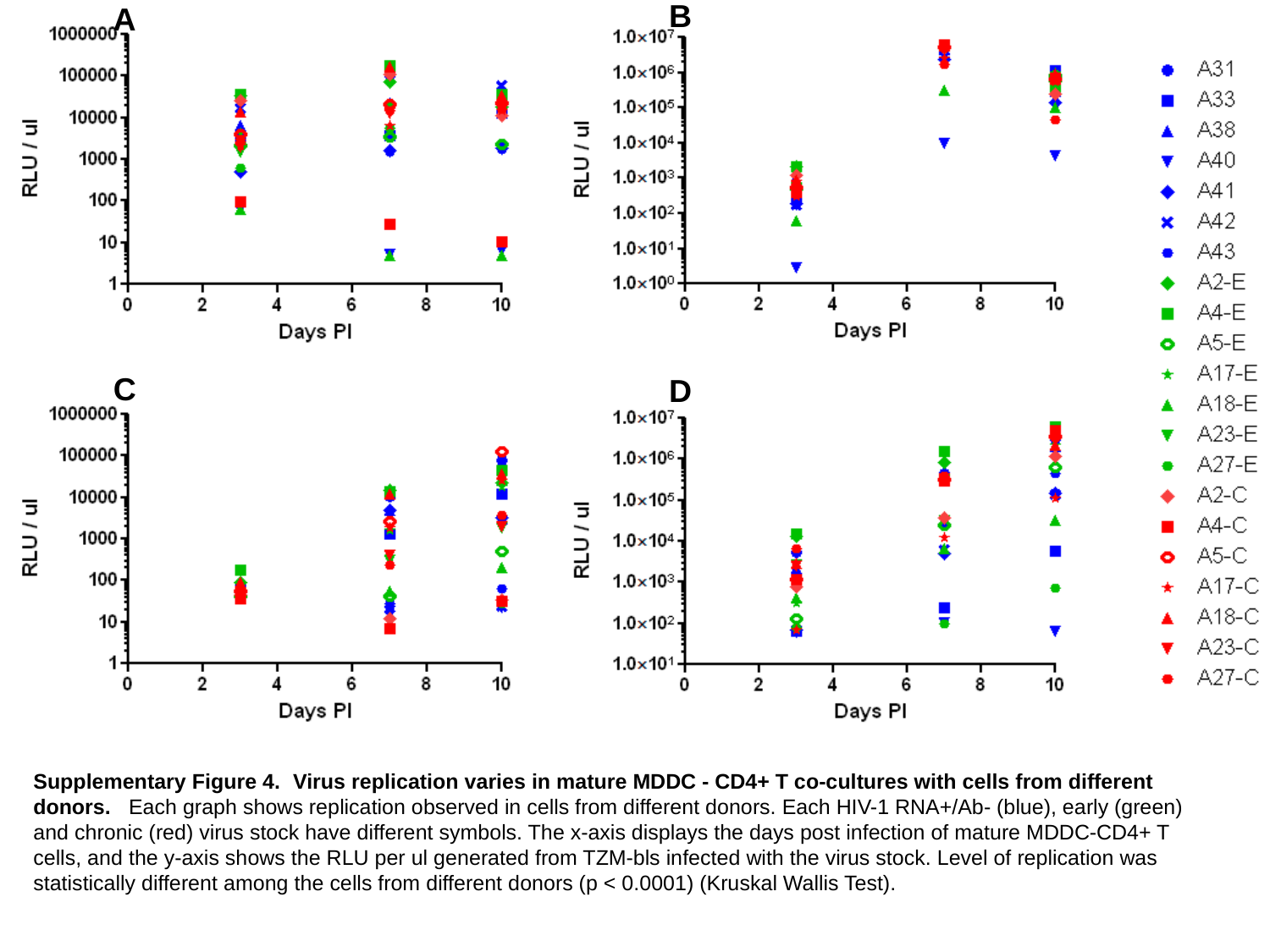

B
A
C
D
Supplementary Figure 4. Virus replication varies in mature MDDC - CD4+ T co-cultures with cells from different donors. Each graph shows replication observed in cells from different donors. Each HIV-1 RNA+/Ab- (blue), early (green) and chronic (red) virus stock have different symbols. The x-axis displays the days post infection of mature MDDC-CD4+ T cells, and the y-axis shows the RLU per ul generated from TZM-bls infected with the virus stock. Level of replication was statistically different among the cells from different donors (p < 0.0001) (Kruskal Wallis Test).

## Slide 5
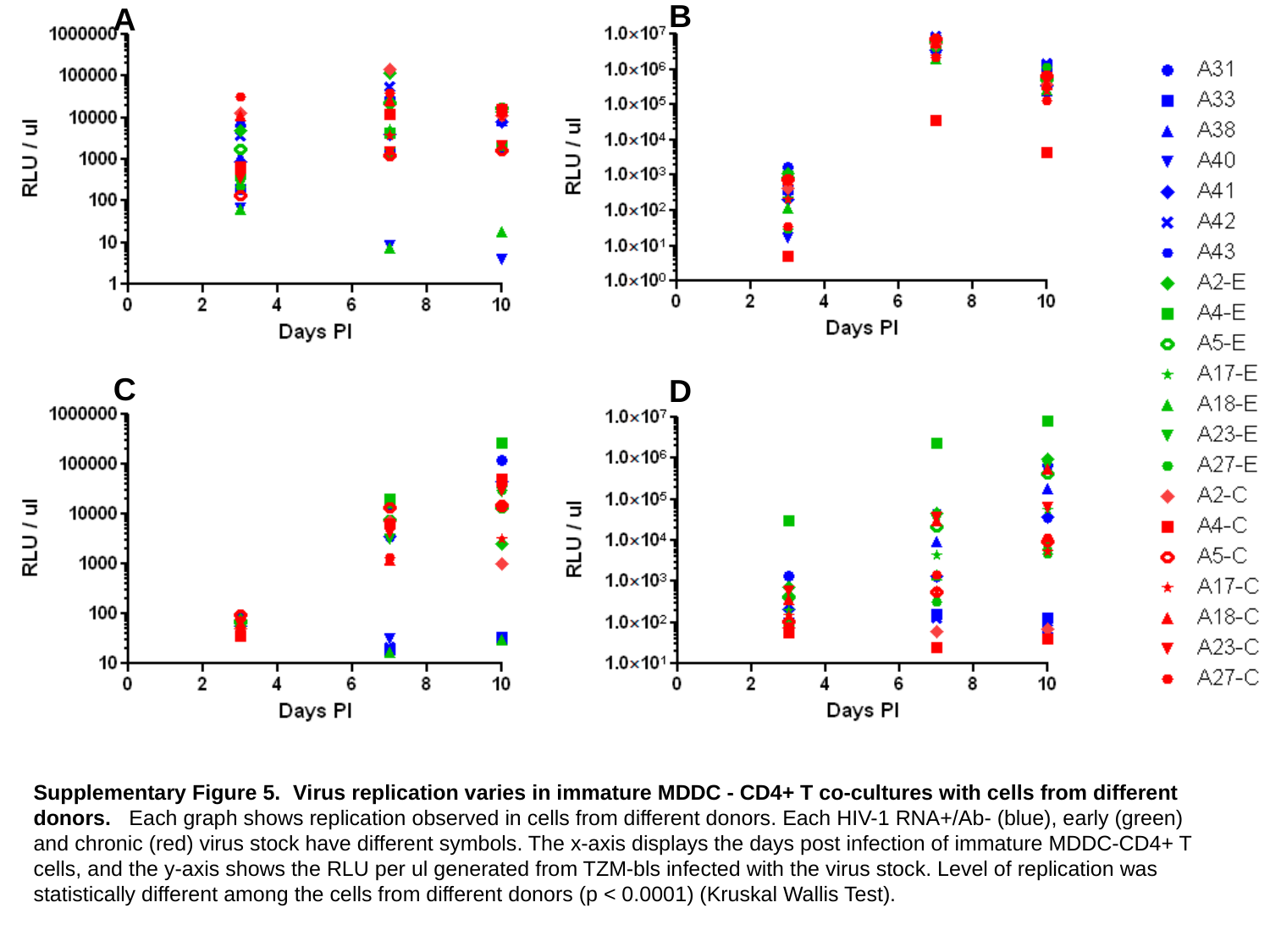

B
A
C
D
Supplementary Figure 5. Virus replication varies in immature MDDC - CD4+ T co-cultures with cells from different donors. Each graph shows replication observed in cells from different donors. Each HIV-1 RNA+/Ab- (blue), early (green) and chronic (red) virus stock have different symbols. The x-axis displays the days post infection of immature MDDC-CD4+ T cells, and the y-axis shows the RLU per ul generated from TZM-bls infected with the virus stock. Level of replication was statistically different among the cells from different donors (p < 0.0001) (Kruskal Wallis Test).

## Slide 6
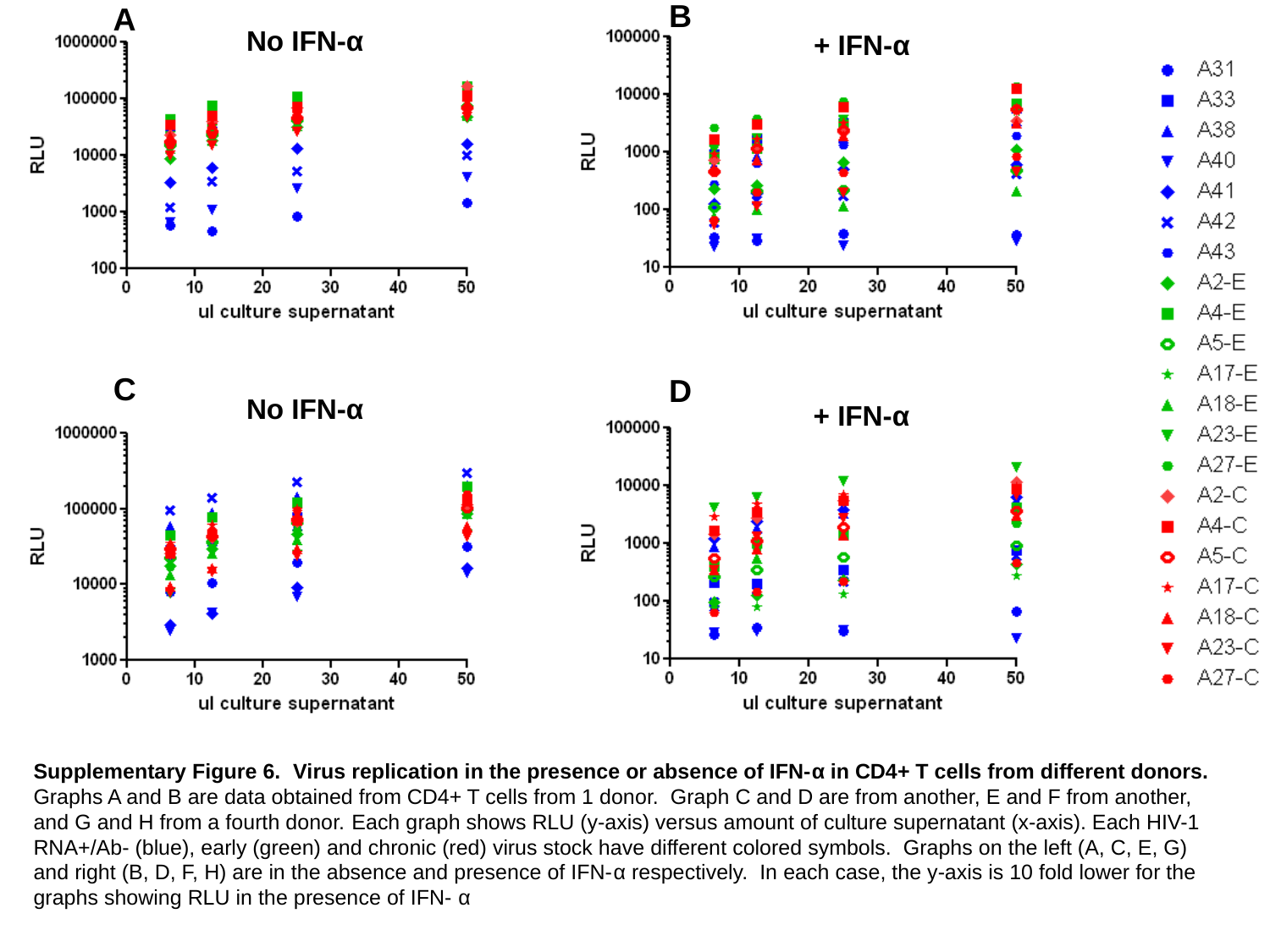

B
A
No IFN-α
+ IFN-α
C
D
No IFN-α
+ IFN-α
Supplementary Figure 6. Virus replication in the presence or absence of IFN-α in CD4+ T cells from different donors. Graphs A and B are data obtained from CD4+ T cells from 1 donor. Graph C and D are from another, E and F from another, and G and H from a fourth donor. Each graph shows RLU (y-axis) versus amount of culture supernatant (x-axis). Each HIV-1 RNA+/Ab- (blue), early (green) and chronic (red) virus stock have different colored symbols. Graphs on the left (A, C, E, G) and right (B, D, F, H) are in the absence and presence of IFN-α respectively. In each case, the y-axis is 10 fold lower for the graphs showing RLU in the presence of IFN- α

## Slide 7
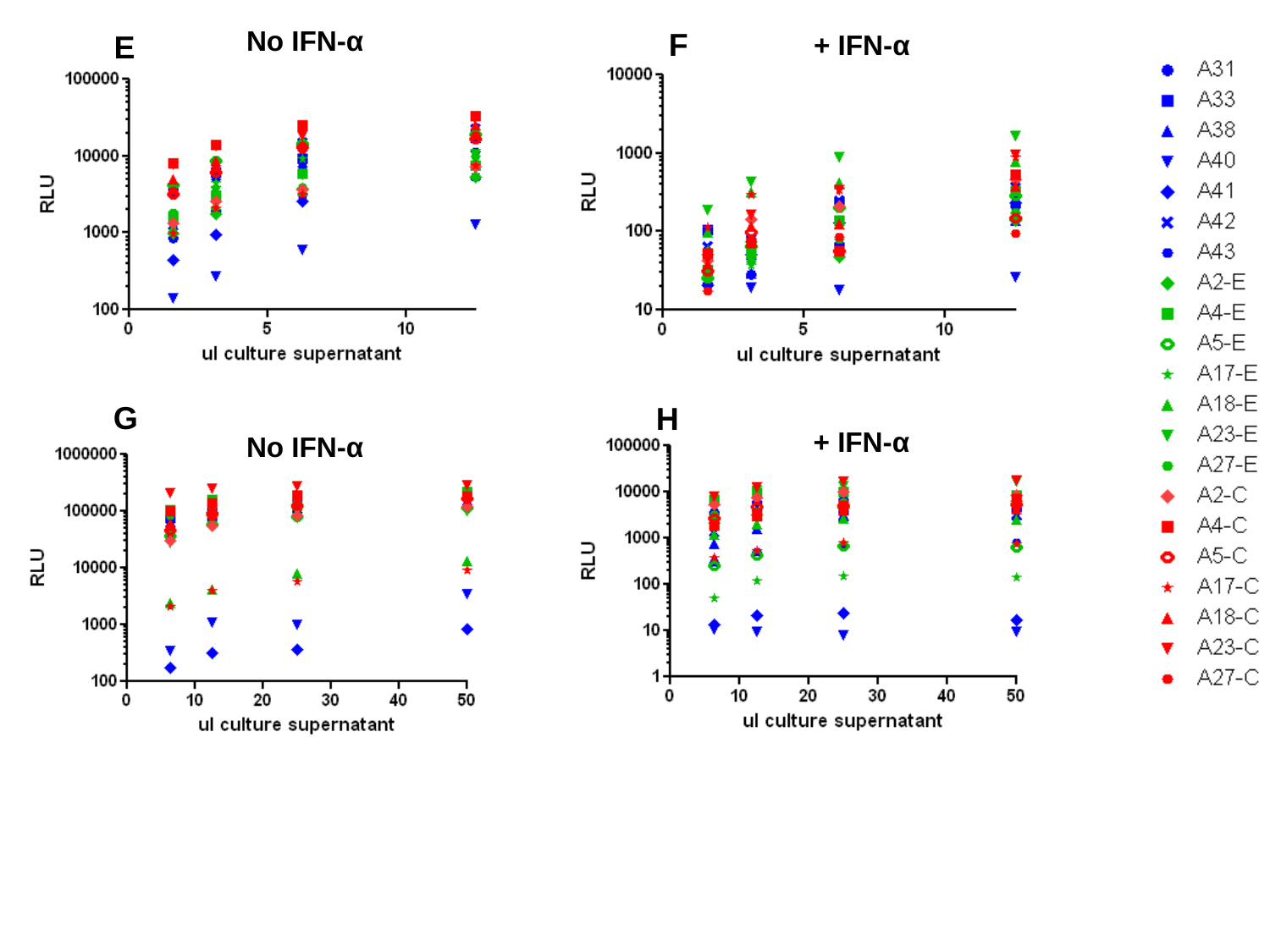

No IFN-α
F
E
+ IFN-α
G
H
+ IFN-α
No IFN-α

## Slide 8
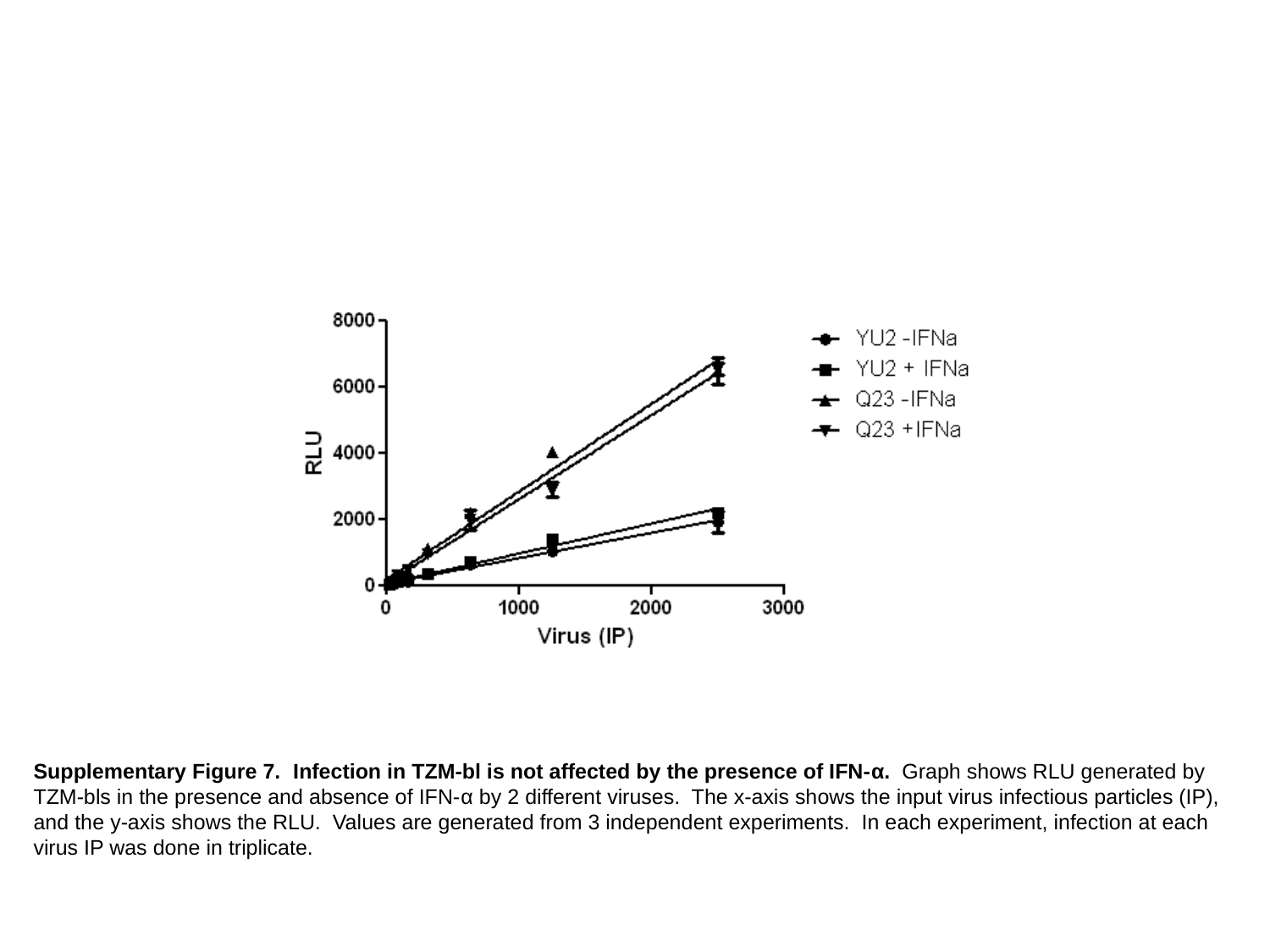

Supplementary Figure 7. Infection in TZM-bl is not affected by the presence of IFN-α. Graph shows RLU generated by TZM-bls in the presence and absence of IFN-α by 2 different viruses. The x-axis shows the input virus infectious particles (IP), and the y-axis shows the RLU. Values are generated from 3 independent experiments. In each experiment, infection at each virus IP was done in triplicate.
